# Integrative Advances in Equine Genomics From Reference Assemblies to Evolutionary History and Key Traits

**DOI:** 10.1111/eva.70283

**Published:** 2026-06-17

**Authors:** Ying Lu, Ruoshan Ma, Bo Wang, Jiao Wu, Yuqing Chong, Zhendong Gao, Weidong Deng

**Affiliations:** ^1^ Faculty of Animal Science and Technology Yunnan Agricultural University Kunming China; ^2^ State Key Laboratory for Conservation and Utilization of Bio‐Resource in Yunnan Kunming China

**Keywords:** genetic diversity, horse genome, key traits, origin and evolution, reference assembly

## Abstract

Horses are major domestic animals and cultural symbols that have accompanied humans for millennia. They underpin transport, agriculture, warfare and sport, and also provide a model for studying domestication, complex traits and adaptive evolution. Recent work in equine genomics has now generated a much richer picture of how these roles are grounded in the genome. This review brings together advances in several connected areas: the construction and refinement of reference assemblies; genomic reconstructions of origin, domestication and dispersal; global and regional patterns of genetic diversity; and the molecular basis of key traits such as athletic performance, coat colour, body size, environmental adaptation and inherited myopathies. The transition from EquCab1.0/2.0 to EquCab3.0 and a complete Y‐chromosome sequence illustrates how long‐read and Hi‐C/T2T data improve genome completeness and the representation of complex regions. On this foundation, high‐coverage resequencing of ancient and modern horses has clarified the geographical core of domestication in the Volga–Don region, the Bronze Age replacement of earlier domestic lineages and the long‐term impact of human management on behaviour, conformation and mobility. Comparative analyses of mitochondrial DNA, Y‐chromosomal haplotypes and autosomal runs of homozygosity further reveal a combination of diverse maternal lineages, highly constrained paternal lineages and breed‐specific inbreeding histories. Against this background, studies of representative traits show how association signals, functional experiments and clinical evidence can be linked to practical tools for breeding and health management, for example through *MSTN*‐guided performance profiling, *EPAS1*‐based altitude adaptation and molecular tests for *GYS1*, *SCN4A*, *PPIB* and *MYH1*. We conclude by considering how telomere‐to‐telomere assemblies, pangenome resources, improved structural‐variant detection and closer integration between population genomics and functional studies may support conservation, health surveillance and molecular breeding in diverse horse populations.

**Abbreviations**
ACAN: aggrecan
ARNT: aryl hydrocarbon receptor nuclear translocator
ASIP: agouti signalling protein
CKM: creatine kinase, M‐type
CKY: chakouyi
COX4I2: Cytochrome C oxidase subunit 4I2
DMRT3: doublesex and Mab‐3 related transcription factor 3
EDNRB: endothelin receptor type B
EPAS1: endothelial PAS domain protein 1
FAO: Food and Agriculture Organization of the United Nations
GABPA: GA binding protein transcription factor subunit alpha
GSDMC: gasdermin C
GWAS: genome‐wide association study
GYS1: glycogen synthase 1
HERDA: hereditary equine regional dermal asthenia
HMGA2: high mobility group AT‐Hook 2
HPS5: HPS5 biogenesis of lysosomal organelles complex 2 subunit 2
HSPA1A: heat shock protein family A (Hsp70) member 1A
HYPP: Hyperkalemic periodic paralysis
KIT: KIT proto‐oncogene, receptor tyrosine kinase
LASP1: LIM and SH3 protein 1
MC1R: melanocortin 1 receptor
MITF: melanocyte inducing transcription factor
MSTN: myostatin
MYL2: myosin light chain 2
NFKBIA: NFKB inhibitor alpha
NRAP: nebulin related anchoring protein
OLWS: overo lethal white syndrome
PAPSS2: 3″‐phosphoadenosine 5″‐phosphosulfate synthase 2
PAX3: paired box 3
PDK4: pyruvate dehydrogenase kinase 4
PMEL: premelanosome protein
PPARGC1A: PPARG coactivator 1 alpha
PSSM1: polysaccharide storage myopathy type 1
RFWD3: ring finger and WD repeat domain 3
ROH: homozygosity
SA: skeletal atavism
SCN4A: sodium voltage‐gated channel alpha subunit 4
SHOX: SHOX homeobox
SLC36A1: solute carrier family 36 member 1
SLC45A2: solute carrier family 45 member 2
SOCS4: suppressor of cytokine signaling 4
STX17: syntaxin 17
SVs: structural variants
T2T: telomere‐to‐telomere
TBX4: T‐Box transcription factor 4
TRHR: thyrotropin releasing hormone receptor
ZFAT: zinc finger and AT‐Hook domain containing
ZFPM1: zinc finger protein, FOG family member 1

## 1 Introduction

Equids, particularly the domestic horse, have played a central and distinctive role in animal domestication and the development of human civilization (Klecel and Martyniuk 2021; Outram et al. 2009). Since the Bronze and Iron Ages, the development of riding and draught technologies has transformed long‐distance transport and the mobility of warfare, reshaping steppe civilizations, trade networks and territorial configurations (Librado et al. 2016; Outram et al. 2009). In this sense, the horse is not only an important domestic species, but also a representative model for understanding how human mobility, cultural innovation and animal evolution became linked during domestication.

In the modern era, horses contribute in diverse ways, including racing and equestrian sports, mounted police forces and pastoral production, cultural tourism and wellness and equine‐assisted therapy, and these activities together continue to reshape perceptions of their social and economic roles (Klecel and Martyniuk 2021; Mendona et al. 2019; Stern and Chur‐Hansen 2019). This broad social role ultimately rests on genetic foundations: the status of the domestic horse cannot be separated from the long‐term evolutionary processes underlying its origin, domestication and dispersal (Librado et al. 2016; Orlando 2020).

Ancient genomics uses whole‐genome sequencing of temporally and geographically stratified ancient and modern samples to reconstruct the spatiotemporal trajectory from origin through domestication to subsequent dispersal (Fages et al. 2019; Gaunitz et al. 2018; Librado et al. 2021). Current evidence indicates that although early steppe cultures already exploited and tamed wild horses, the main ancestral source of modern domestic horses was more likely concentrated in the Volga–Don region of the western Eurasian steppe.

From the Bronze Age onward, this lineage spread rapidly across Eurasia along military, pastoral and commercial routes (Librado et al. 2021; Outram et al. 2009). Over subsequent millennia, artificial selection under culturally and functionally differentiated regimes (war, herding, racing) imposed distinct pressures on behaviour, exercise metabolism and body conformation, creating a complex domestication landscape shaped by interactions between genomes, technological innovations and social functions (Fages et al. 2019; Librado et al. 2017).

Globally, the rich and diverse pool of local horse genetic resources is facing declining numbers and an imbalance between conservation and utilization against the backdrop of modern agricultural and industrial transformation (Fages et al. 2019; Petersen et al. 2013). This situation highlights the value of genome‐centred evidence as a basis for resource inventory, targeted conservation and efficient directional breeding (Raudsepp et al. 2019; Wade et al. 2009). Whole‐genome sequencing is now widely used to characterise genetic diversity, reconstruct origins and evolutionary history, map functional loci for important traits and assess disease risk, thereby supporting the development of reproducible molecular‐breeding workflows (Librado et al. 2017, 2021; Raudsepp et al. 2019).

Sequencing technologies themselves have progressed from first‐generation Sanger sequencing to second‐generation short‐read high‐throughput platforms (such as Illumina), and more recently to third‐generation single‐molecule long‐read sequencing (PacBio, Nanopore). These advances have improved the continuity and accuracy of reference genomes in many species and expanded the range of complex repeat regions and structural variants that can be resolved (Gao et al. 2024; Liu 2025; Luo et al. 2025). Their significance, however, extends beyond technical improvement. By revealing previously inaccessible regions and variant types, long‐read and chromosome‐scale assemblies provide new entry points for interpreting trait evolution, regulatory change and genetic disease. In parallel, comparative genomic frameworks that systematically link phenotype to genotype are becoming increasingly mature. These frameworks provide conceptual guidance for connecting equine genomic resources to trait mapping and functional interpretation (Hilgers and Hiller 2025).

In horses, the reference genome has evolved from EquCab1.0/2.0 to EquCab3.0, which integrates short reads, long reads and Chicago/Hi‐C proximity ligation data, and further towards telomere‐to‐telomere (T2T) assemblies. This progression has substantially improved assembly and annotation quality and provided more reliable anchor points for studies of breed comparison, trait discovery and population evolution (Kalbfleisch et al. 2019; Raudsepp et al. 2019; Wade et al. 2009).

This review brings together archaeological, ancient DNA and modern population genomic evidence to provide an integrated perspective on equine genomics. We first summarise milestone advances in reference genome construction and current views on *Equus* origins and evolutionary history, and then outline global patterns of genetic diversity among horse breeds and genomic findings on key economic traits, including athletic performance, coat colour and body size.

These strands are later brought into a common analytical framework that links reference and breed‐specific assemblies, population structure and ancient genomic time series, and traits‐associated loci and selection signatures, with an emphasis on breeding and conservation strategies that balance genetic diversity, health and performance. The final sections consider how recent developments in long‐read assembly, structural‐variant analysis and multi‐omics integration can move equine genomics from resource description towards mechanistic understanding and practical molecular breeding, and how these developments may provide conceptual and technical guidance for future work.

## 2 Construction and Iterative Improvement of Equine Reference Genomes

As whole‐genome sequencing became widely available in domestic animals, the horse was one of the first livestock species to obtain a chromosome‐level reference assembly. Subsequent work has focused on refining the Twilight‐based EquCab series and adding representative assemblies from multiple breeds and wild relatives. Together, these resources document the transition from EquCab1.0 to EquCab3.0, extend coverage with breed‐specific and pangenome assemblies, and provide the genomic framework for analysing population history and complex traits. Beyond their technical value, these reference genomes also provide the coordinate system through which equine domestication, breed formation, artificial selection and trait variation can be interpreted.

### 2.1 Evolution of the Twilight‐Based Reference (EquCab1.0–3.0)

The main reference genomes used in current equine research are EquCab1.0, EquCab2.0 and EquCab3.0. All three assemblies were generated from the same Thoroughbred mare, Twilight, but they differ substantially in sequencing strategies, assembly quality and functional completeness. With advances in sequencing technologies, these versions have been updated as methods have improved, moving from an initial draft genome to a high‐quality reference and more recently towards more complete chromosome‐scale assemblies.

In 2007, a team at the Broad Institute produced the first equine genome draft, EquCab1.0, based on the genome of the Thoroughbred mare Twilight. Using approximately 6.8× Sanger whole‐genome shotgun data in combination with BAC library information, they obtained a chromosome‐level assembly in which roughly 84% of the sequence was anchored to 31 pairs of autosomes and the X chromosome, thereby providing initial coordinates and an annotation framework for subsequent EquCab reference genomes (Horse Genome Project, Broad Institute; UCSC equCab1 release). EquCab1.0 was mainly released via project websites and databases and was not accompanied by a dedicated publication. Although its assembly continuity and missing sequence rate left considerable room for improvement, this version nonetheless established the basic scale and breed representativeness of the domestic horse reference genome. It also provided the first framework for comparative genomic analyses and for linking genomic regions with traits shaped by domestication and breed development.

EquCab2.0 continued to use Twilight as the source individual. Building on the original ~6.8× Sanger whole‐genome data, the authors incorporated BAC end pairs and radiation hybrid and FISH physical maps to reassemble and re‐anchor the EquCab1.0 scaffolds, thereby markedly improving assembly continuity and chromosomal placement accuracy. EquCab2.0 has a total assembly size of about 2.68 Gb, of which roughly 2.33 Gb are non‐N bases, with a contig N50 of ~0.11Mb and a scaffold N50 of ~46–47 Mb. These metrics made the horse one of the first large domestic animals to possess a comparatively high‐quality reference genome and provided an essential foundation for downstream comparative genomics, SNP chip development and genetic improvement studies (Wade et al. 2009). Subsequent assessments using BUSCO and related tools indicated that EquCab2.0 covers about 99% of the core conserved mammalian gene set, sufficient to support many functional genomics applications, although limitations remained in resolving complex repeats, refining gene structures and capturing some functionally relevant but incomplete regions.

With the advent of long‐read sequencing and three‐dimensional genome technologies, EquCab2.0 served as the coordinate framework for a comprehensive reassembly that integrated Sanger reads, Illumina short reads, PacBio long reads and Chicago/Hi‐C data to generate the third‐generation reference genome EquCab3.0. Without markedly changing the overall genome size (non‐N sequence ~2.41 Gb), EquCab3.0 increased the contig N50 to the megabase scale (~4.5 Mb), representing nearly a 40‐fold improvement over EquCab2.0, and achieved a scaffold N50 of approximately 86–87 Mb, resulting in more complete and continuous chromosomal sequences. BUSCO evaluations showed that EquCab3.0 contains about 99.7% complete genes and exhibits substantially reduced fragmentation and missingness (Kalbfleisch et al. 2019). Compared with EquCab1.0/2.0, EquCab3.0 places stronger emphasis on limiting systematic errors in downstream comparative genomics. Improved assembly of centromeric and telomeric flanking regions, GC‐biassed segments and repeat‐rich regions directly diminishes technical peaks in population differentiation and selection scans, and it also provides a more reliable reference framework for structural variant detection, transcript structure analysis and functional element annotation. Thus, EquCab3.0 not only improved reference quality, but also strengthened the ability to connect genomic variation with population history, artificial selection and complex trait architecture. In the same year, a more complete Y chromosome assembly was generated from deeply sequenced individuals, filling a major gap in the sex chromosome complement and bringing male reproductive candidate gene clusters and their associated variants into a testable hypothesis space (Janečka et al. 2018).

### 2.2 Breed‐Specific Assemblies and Emerging Equine Pangenome Resources

Building on these latest reference‐genome iterations, Gu and colleagues constructed ten chromosome‐level, three‐dimensional genomes representing distinct domestic horse breeds. Using a multi‐assembly comparative strategy, they systematically characterized structural variation and identified thousands of insertion segments that are absent from the traditional reference genome. This work marks an important shift from a single‐reference view of the horse genome towards a variation‐aware framework that can better represent breed‐level diversity. They further proposed an Equus‐specific mechanism in which structural variants enriched for long interspersed nuclear elements reshape gene regulatory networks, thereby providing an important conceptual framework for developing equine pangenome resources that more fully capture natural genomic diversity (Gu et al. 2023).

In parallel with continuous upgrades to the Thoroughbred‐based reference, researchers have begun to generate whole‐genome sequences for multiple representative breeds and to map these sequences onto the EquCab2.0/3.0 backbone, shifting from reliance on a single reference towards a complementary architecture that combines a core reference with breed‐specific increments. For example, the Quarter Horse, a globally important performance breed, was sequenced using second‐generation technologies. After aligning reads to the reference genome, the authors *de novo* assembled unmapped reads and identified approximately 19.1 Mb of Quarter Horse–specific sequence, suggesting that non‐reference sequence harbours candidate functional regions associated with speed and athletic performance (Doan et al. 2012). This work illustrates that sequence lying outside the canonical reference is not merely sequencing noise but can represent loci enriched for breed‐ and trait‐associated variation. Incorporating such sequences into subsequent QTL co‐localization and candidate gene analyses helps to reduce information loss and improve the interpretability of mapping results.

Mongolian horses are renowned for endurance and environmental robustness. *De novo* sequencing of this breed yielded a more refined genomic map for both Mongolian horses and Przewalski's horses, and produced a more complete equine Y chromosome sequence, providing critical evidence for phylogenetic reconstruction, admixture history and adaptation to cold, high‐altitude environments (Huang et al. 2014). Following the release of EquCab3.0, strategies that combine alignment to a common reference with breed‐specific assemblies have been applied to Quarter Horses, Mongolian horses, Icelandic horses and other breeds. By identifying breed‐specific sequence segments and structural variants, these efforts both compensate for blind spots of a single reference in complex and highly polymorphic regions and provide new clues for mapping target traits (Doan et al. 2012; Huang et al. 2014; Librado et al. 2021). On this basis, newly released haplotype‐resolved reference genomes for Dutch Warmblood and Friesian horses in 2025 demonstrate that long‐read sequencing combined with trio‐binning can preserve EquCab3.0‐level continuity while substantially enhancing the resolution of breed‐specific structural variations and allelic polymorphisms. These assemblies constitute key incremental resources for building an equine pangenome (Steensma et al. 2025). Meanwhile, a new long‐read‐based Przewalski's horse genome has reached reference quality, with overall metrics comparable to EquCab3.0, providing an updated framework for conservation genomics in wild equids (Faulk 2025). Together, these resources indicate that equine genomics is moving from reference improvement alone towards a pangenome‐based model that integrates domestication history, wild‐relative conservation, breed‐specific adaptation and molecular breeding.

### 2.3 Technical Implications for Downstream Analyses

**FIGURE 1:**
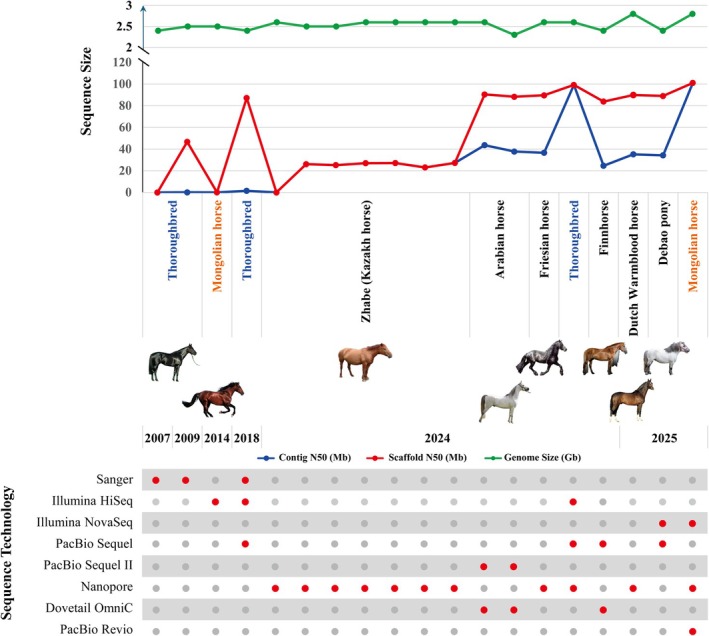
Schematic timeline of iterative improvements in assembly quality and sequencing technologies for the domestic horse reference genome. The upper panel summarizes changes in genome size, contig N50 and scaffold N50 across representative horse genome assemblies. The lower panel shows the major sequencing and scaffolding technologies used in each assembly. This figure is intended to provide a visual overview of technological transitions and assembly‐quality improvements, whereas detailed assembly information is provided in Table 1.

These developments show that the domestic horse reference genome has been updated repeatedly in both assembly quality and sequencing platforms. The main assembly metrics and sequencing strategies for each version are summarized in Table 1, and their temporal progression is illustrated in Figure 1. Considered side by side, the datasets in Table 1 and Figure 1 highlight a clear improvement in the continuity and completeness of the horse reference genome with the introduction of long reads, proximity ligation and chromosome conformation capture technologies. Improvements in contig N50, structural variant resolution and the assembly of complex repeats, in particular, have markedly reduced technical noise in subsequent trait mapping and population comparisons.

**TABLE 1:** Overview of assembly statistics and major sequencing strategies for different versions of the domestic horse reference genome.

Breed	Genome version	Sequencing technologies	Sequence size (Gb)	Assembling evaluation metrics			References
				Contig N50 (Mb)	Scaffold N50 (Mb)	BUSCO (%)	
Thoroughbred	EquCab1.0	Sanger	~2.4	NA	NA	NA	Broad Institute, 2007
	EquCab2.0	Sanger	2.7	0.11	46.7	NA	Wade et al. (2009)
	EquCab3.0	Sanger; Illumina HiSeq; PacBio	2.5	4.5	86	98.8	Kalbfleisch et al. (2019)
Mongolian horse	Ajinai1.0	Illumina HiSeq	2.4	0.04	0.0563	—	Huang et al. (2014)
Zhabe (Kazakh horse)	2H	Nanopore	2.6	25.3	25.3	93.2	Assanbayev et al. (2024)
	7H	Nanopore	2.5	26.1	26.1	91.8	
	9H	Nanopore	2.5	25.2	25.2	—	
	16H	Nanopore	2.6	27	27	94.9	
	25H	Nanopore	2.6	27.1	27.1	94.8	
	30H	Nanopore	2.6	23.1	23.1	94.9	
	57H	Nanopore	2.6	27.2	27.2	95.1	
Arabian horse	mEquCab1.pat	PacBio Sequel II HiFi; Dovetail OmniC	2.6	43.6	90.4	—	GCA_036426135.1
	mEquCab1.mat	PacBio Sequel II HiFi; Dovetail OmniC	2.3	37.7	88.3	—	GCA_036418255.1
Friesian horse	EquCab_Friesian_WUR	Nanopore	2.6	36.6	89.6	99.68	Steensma et al. (2025)
Dutch Warmblood horse	EquCab_Warmblood_WUR	Nanopore	2.6	35.2	89.9	99.75	
Debao pony	AGIS_DeBao_1.0	PacBio Sequel; Illumina NovaSeq	2.4	34.2	89	96.2	Li et al. (2025)
Thoroughbred	TB‐T2T	Illumina; Oxford Nanopore PromethION; PacBio Sequel	2.8	99.2	99.2	99.1	GCA_041296265.1
Finnhorse	EquCab_Finn	PacBio; Omni‐C	2.4	24.6	83.8	96.3	Pokharel et al. (2024)
Mongolian horse	T2T‐horse1.0	Illumina NovaSeq; Oxford Nanopore PromethION; PacBio Revio	2.8	101	101	98.8	Liu, Wang, et al. (2025)

*Note:* EquCab1.0 was released only via project websites and databases, and systematic N50 and BUSCO evaluations have not been reported; the corresponding cells are therefore denoted as ‘NA’.

On this foundation, equine genomics has increasingly followed a technical strategy that centres on a single high‐quality reference genome and is complemented by high‐quality assemblies from multiple breeds, bringing together a core reference with breed‐specific genomic increments. The former provides a common coordinate system for cross‐study comparison, whereas the latter captures sequence diversity and structural variation that are difficult to represent in a single reference. This combined strategy helps bridge population history, functional variation and applied trait analysis within a shared genomic framework. As a whole, these resources now provide the foundation for analyses of complex traits such as athletic performance, coat colour and body size (Kalbfleisch et al. 2019; Librado et al. 2021; Wade et al. 2009).

## 3 Genomic Sequencing Approaches to Unravel *Equus* Evolutionary History

Genomic datasets have become central to reconstructing the origin, dispersal, and domestication of *Equus*. In recent years, work has moved from studies based on a few molecular markers to time‐stratified whole genomes, from single‐step domestication scenarios to multi‐phase models centred on the DOM2 lineage, and from mainly qualitative narratives to a more formal three‐layered framework for *Equus* evolutionary history.

### 3.1 From Limited Markers to Time‐Stratified Ancient Genomes

When addressing the origin, dispersal and domestication of *Equus*, research has gradually shifted from a focus on archaeological material combined with a limited set of molecular markers to an approach based on time‐stratified whole‐genome sequences from ancient and modern samples (Librado et al. 2017; Orlando et al. 2013; Schubert et al. 2014). This shift has enabled recalibration of molecular clocks and re‐estimation of divergence tempos among lineages. It also allows researchers to jointly model effective population size, migration and replacement history, and selection trajectories along a single time axis, which helps disentangle and quantify the environmental gradients and human management practices acting on the genome (Fages et al. 2019; Librado et al. 2021; Orlando et al. 2013). Within this framework, comparative analyses of ultra‐ancient genomes have pushed the divergence among the lineages leading to modern horses, zebras and donkeys back to roughly 4.5–4.0 Ma, substantially earlier than traditional estimates based on limited markers (Orlando et al. 2013).

Ultra‐ancient samples thereby extend the time window of lineage divergence back to the Middle Pleistocene and provide direct genomic evidence for early *Equus* evolution (Orlando et al. 2013). The most recent analyses of mitochondrial genomes from *Equus mosbachensis* recovered at the open‐air Middle Pleistocene site of Schöningen in Germany place a major divergence event within *Equus* at around 570 thousand years ago and demonstrate that ancient DNA up to ~300 thousand years old can still be recovered even from non‐cave, non‐permafrost settings. These findings provide a new deep‐time baseline and a methodological template for studying early *Equus* evolution (Weingarten et al. 2025). In this way, ancient genomes help connect deep‐time evolutionary history with the more recent processes that shaped domestic horses and their wild relatives.

### 3.2 Multi‐Phase Domestication, DOM2 and Regional Lineages

Time‐series studies have shown that Eurasian Late Pleistocene to Bronze Age horse populations record a complex history of genetic continuity, turnover and partial replacement rather than a simple linear transition to modern domestic horses. They also show that early domestication‐phase selection focused more strongly on behavioural regulation and stress‐response pathways, with later expansion of selection targets to more conspicuous phenotypic modules such as coat colour and body conformation (Fages et al. 2019; Librado et al. 2017; Schubert et al. 2014). Within this framework, a time‐series scan of 266 trait‐associated loci across ancient and modern samples has further resolved key nodes of this progressive selection process: a behaviour‐associated locus near *ZFPM1* (zinc finger protein, FOG family member 1) shows strong positive selection signals from around 5000 years ago, whereas the *GSDMC* (gasdermin C) locus, associated with body conformation and spinal morphology, has experienced intense selection since about 4750 years ago and reached high frequency by approximately 4150 years ago (Librado et al. 2021; Liu, Jia, et al. 2025). Functional experiments indicate that *GSDMC* genotypes are significantly associated with trunk configuration in horses and also affect spinal anatomy, motor coordination and muscle strength in mouse models (Frantz 2025; Liu, Jia, et al. 2025). Considered as a whole, these lines of evidence point to selection for standing stability and load‐bearing capacity as a key genetic step during the domestication bottleneck, enabling the rapid rise of highly mobile, rideable horses. In temporal terms, they outline a sequence in which behavioural and neural traits were targeted first, followed by conformation and locomotor performance (Frantz 2025; Liu, Jia, et al. 2025).

Horse domestication and dispersal did not proceed linearly along a single lineage, but instead involved multiple domestication attempts and lineage replacements. Time‐resolved data from the Bronze and Iron Ages support the staged selection scenario described above, with behavioural and neural traits targeted earlier and conformation and coat colour traits selected later (Fages et al. 2019; Librado et al. 2017). Larger ancient genomic time series further indicate that around 5 ka BP there were domesticated horse lineages in Iberia and Siberia that are now extinct. These early domesticated horses contributed very little to the genetic background of modern domestic horses, suggesting that some early domestication lineages did not persist as components of the main modern domestic horse gene pool (Fages et al. 2019).

As ancient genomic coverage has expanded, the geographic location of the main ancestral source of modern domestic horses has converged on a consistent picture: the lower Volga–Don steppe in western Eurasia has been identified as the origin core of the domestic horse lineage that subsequently became globally dominant. The unified modern domestic lineage arising from this region is commonly termed the second domestic horse lineage (DOM2) in the literature (Librado et al. 2021; Librado and Orlando 2021). Inferred demographic histories suggest that ancestral populations in this region first expanded into Anatolia, the lower Danube and Central Asia around 4200–4000 BP, and between 3500 and 3000 BP rapidly replaced local horse populations across Eurasia, thereby establishing a unified backbone of domestic horse ancestry (Librado et al. 2021). A recent pan‐Eurasian synthesis further shows that systematic reproductive control emerged around ca. 2200 bce, as reflected by increased inbreeding and shortened generation times. At the same time, the migration trajectories of steppe human groups were not perfectly synchronous with horse dispersal, implying that technological and institutional innovations (such as riding, driving and husbandry systems) acted as partly independent drivers in the spread of domestic horses (Librado et al. 2024).

In the eastern Eurasian steppe, the Botai cultural sites of northern Central Asia (ca. 5.5–5.0 ka BP) were long considered prime candidates for an early centre of horse domestication because of the large quantities of horse bones and milk fat residues recovered there. Whole‐genome sequencing of horse bones from these sites has shown that the so‐called Botai domestic horses form a clade with modern Przewalski's horses, distinct from the unified modern domestic lineage DOM2. In other words, Przewalski's horses are not enduring relics of a never‐domesticated wild horse lineage, but rather descendants of early human‐managed horses that returned to the wild over subsequent millennia (Gaunitz et al. 2018). This finding substantially revises how relationships between wild, domestic and feral horse populations are understood and further supports the view that horse domestication involved multiple regional attempts, only some of which ultimately contributed to the main modern domestic horse gene pool.

Regional lines of evidence provide important complements to this global narrative. In China, archaeogenomic work has produced a high‐quality genome assembly for the extinct *Equus sussemionus*, revealing that this species survived into the Bronze Age but shows no signals of domestication. Its extinction time has been pushed back by roughly 8500 years, indicating persistence into the Late Holocene at around 3.5 ka BP and substantially extending the time span over which relationships between wild, domestic and feral horse populations can be traced (Cai et al. 2022). Incorporation of such regional time series makes the three‐stage process linking the origin core, the main dispersal routes and the replacement of local horse populations more amenable to quantitative analysis and formal hypothesis testing (Librado et al. 2021; Vanhorn and Morris 2021).

### 3.3 Methodological Advances and a Three‐Layered Framework

On the methods side, stricter radiocarbon dating and stratigraphic constraints, alignment and variant‐calling pipelines that explicitly model ancient DNA damage patterns, capture‐based targeted sequencing, and statistical frameworks for integrating ultra‐low‐coverage genomes (including ancient‐sample‐specific error models and treatments of incomplete lineage sorting) have all been developed in recent years. As a result, these advances have reduced systematic biases in temporal inference and lineage reconstruction (Fages et al. 2019; Orlando et al. 2013; Schubert et al. 2014). Their value is not only methodological; they make it possible to place genetic change, demographic turnover and selection within a more reliable archaeological and chronological context. When combined with modern population genomic data and functional evidence on behaviour and stress responses, metabolic traits and performance phenotypes, these ancient genomic analyses provide multi‐layered cross‐validation from lineage history through to functional pathways (Fages et al. 2019; Gaunitz et al. 2018; Librado et al. 2021). It is important to emphasize, however, that current time‐stratified ancient genomic datasets still suffer from uneven temporal and geographic sampling, variable DNA preservation and limited numbers of ultra‐ancient specimens. Lineage relationships for several critical periods and regions remain uncertain and will require expanded sample sizes and more harmonized analytical workflows across studies (Librado and Orlando 2021; Orlando 2020; Vanhorn and Morris 2021).

**FIGURE 2:**
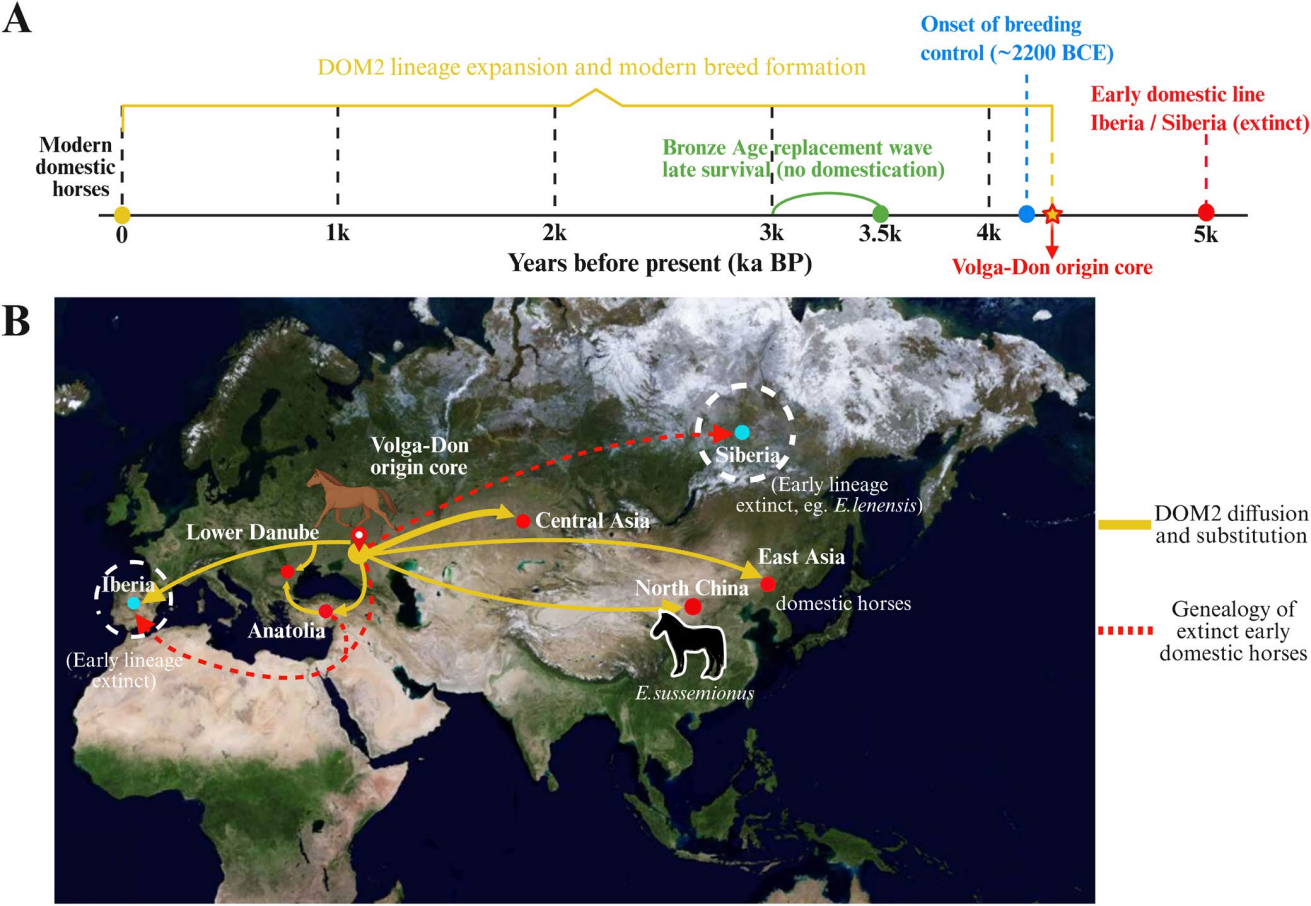
Schematic spatiotemporal overview of *Equus* domestication and dispersal. (A) Based on time‐stratified whole‐genome data from ancient and modern samples, the Late Pleistocene to Holocene domestication and lineage evolution of horses is often described in terms of three main phases: Early domestication attempts and now‐extinct domestic lineages in regions such as Iberia and Siberia; a Bronze Age replacement wave centred on the Volga–Don origin core; and, finally, the unified domestic horse backbone and modern breed diversification shaped by the rapid spread of the DOM2 lineage and intense artificial selection. (B) Ancient genomic and archaeological evidence from across Eurasia support identification of the lower Volga–Don steppe as the origin core of the DOM2 lineage. From this region, domestic horses spread to Anatolia, the Danube basin and Central Asia around 4.2–4.0 ka BP, and between 3.5 and 3.0 ka BP progressively replaced local horse populations across Eurasia. Early domestic lineages in Iberia and Siberia went extinct after the Bronze Age and contributed little to the genetic background of modern domestic horses, whereas in northern China *Equus sussemionus* persisted as an undomesticated wild species until around 3.5 ka BP. The figure provides a visual synthesis of how modern domestic horses emerged from three complementary perspectives: The origin core, dispersal routes and replacement of local populations. The graphical elements are adapted and synthesized from Orlando et al. (2013), Fages et al. (2019), Librado et al. (2017, 2021, 2024), Cai et al. (2022) and related studies.

Current genomic evidence supports a three‐layered view of *Equus* evolution, with an earlier baseline of deep‐time lineage divergence, a Bronze Age wave of unified replacement, and strong artificial selection under modern breeding regimes. In combination, these processes have produced the pattern of high genetic homogeneity at continental scales and pronounced breed specificity at local scales observed in modern domestic horses (Gaunitz et al. 2018; Librado et al. 2021, 2024; Orlando et al. 2013). Against this background, the domestication and dispersal of domestic horses can be viewed as a process unfolding along a temporal axis and spreading across space (Figure 2). This perspective also provides the historical context for subsequent discussion of genetic diversity and local breed resources in modern horses. It further links evolutionary reconstruction with practical questions in conservation, breed management, and the interpretation of trait‐associated variation.

## 4 Genetic Diversity in Horses

Genetic diversity sits at the core of our understanding of breed history, conservation priorities and the genetic architecture of key traits. At a global scale and within China, patterns of diversity reveal how breeds have been shaped by geography and management; cross‐breed catalogues of sequence and structural variants add finer resolution on the distribution of functional alleles; and contrasts among maternal, paternal and autosomal markers highlight different demographic histories. Together, these lines of evidence help to explain how ancient and modern processes have produced the current diversity landscape in domestic horses.

### 4.1 Global Patterns and Chinese Local Resources

Population comparisons based on genome‐wide SNPs and resequencing data consistently indicate that the lineage stratification of modern domestic horses broadly mirrors their geographic origins and breeding histories. Breeds subjected to prolonged closed breeding with small effective population sizes tend to exhibit lower genetic diversity, whereas populations with long‐term introgression of exogenous bloodlines or more complex dispersal histories retain relatively higher diversity (Petersen et al. 2013). At the global scale, statistics from the FAO DAD‐IS database indicate that in 2008 the world's domestic horse population numbered around 58.7 million animals, corresponding to 784 registered breeds, most of which are national or regional local breeds. Europe alone holds more than half of all recognized horse breeds, many of which are maintained in small populations of only a few hundred to a few thousand animals (Khadka 2010). Cross‐period DAD‐IS evaluations consistently show that, among horse breeds, those classified as at risk or already extinct together account for roughly one‐third. The proportion with unknown conservation status has long remained at about one‐third or even more than half, whereas breeds clearly classified as not at risk make up less than one‐third. In practical terms, these patterns point to a landscape in which substantial data gaps and potential risks coexist (Food and Agriculture Organization of the United Nations 2025; Khadka 2010).

Within this context, early reports suggested that China harboured roughly 30 local domestic horse breeds (Chang 2009; Lei et al. 2009; Xie 1987). According to the third national survey of livestock genetic resources and the 2024 Catalogue of Chinese Livestock and Poultry Genetic Resources, the number of registered horse breeds in China now stands at 60, including 29 local breeds, 14 cultivated breeds and 17 introduced and crossbred types (Chen et al. 2025). These local breeds are typically grouped into five major types based on geographic distribution and body conformation: Mongolian, Kazakh, Hequ, southwestern Chinese and Tibetan horses. As a group, they serve as important regional reservoirs of genetic diversity at the eastern end of the Eurasian landmass (Chang 2009; Lei et al. 2009; Xie 1987). Using data from 36 breeds and more than 800 individuals, integrated analyses of F_ST_, phylogenetic trees and genetic distance matrices have further delineated the correspondence between breeds, regions and historical backgrounds, providing a widely used baseline for comparative frameworks organized around lineage, trait and application (Petersen et al. 2013). For Chinese local resources, a recent whole‐genome study of the Baise horse combined resequencing data from seven Chinese and five foreign breeds (236 individuals in total) with population structure and ancestry analyses to clarify its origin and ancestral composition. The results show that Baise horses carry multiple ancestral components consistent with their geographic dispersal routes and breeding history, thereby providing a clear population‐genetic framework for targeted conservation and directional improvement of this local breed (Lin et al. 2024).

With respect to ponies and small‐bodied horses, high‐quality reference genomes for the Debao pony, together with global resequencing data from 64 breeds and 452 individuals, have revealed that East Asian horse populations retain a relatively high proportion of ancient genetic ancestry. These analyses also indicate close paternal relationships between several European pony breeds and Asian ponies, providing new population‐genetic evidence for an eastern paternal origin of European pony lineages (Li et al. 2025). Recent comparative whole‐genome work on the endangered Ningqiang pony further shows a continuous decline in effective population size and identifies candidate genes and selection signals associated with height variation, highlighting the unique value of local pony breeds in both genetic diversity conservation and size‐related improvement (Han et al. 2025).

Beyond China, whole‐genome resequencing of the Mugalzhar horse, a Kazakh breed adapted to the continental steppe environment, has similarly revealed its genetic diversity, demographic history and candidate genes associated with local environmental adaptation (Kassymbekova et al. 2025). Incorporating such breed‐level genomic datasets from Central Asia into comparative analyses will further refine our understanding of how regional management regimes, climate and workload jointly shape the diversity landscape of modern horses.

### 4.2 Genomic Variant Catalogues and Mutational Load

At the level of variant types, cross‐breed maps of sequence and structural polymorphisms are equally important for understanding equine genetic diversity. One study conducted whole‐genome sequencing in six horse breeds representing distinct lineages and systematically detected and annotated SNPs and multiple classes of structural variants (SVs), greatly enriching the catalogue of genetic variation in horses and providing a high‐coverage set of candidate loci for trait mapping and population comparisons. Such inventories that combine multiple breeds and multiple variant types complement the single reference genome and jointly extend the resolution limits for complex regions and structural variation (Abriid et al. 2020). At the population scale, integrating 37 newly sequenced genomes with public datasets for a total of 97 domestic horses, Gu et al. (2023) combined SVs and SNPs in population genomic analyses to screen for candidate regions associated with domestication history and phenotypic differences in size and performance among breeds, and used Thoroughbreds as a case study to explore how structural variants may shape breed‐specific genetic backgrounds. This case provides a representative example of how to interpret equine genetic diversity within an integrated framework linking structural variation, population history and functional traits.

The most recent large‐scale resequencing dataset, encompassing 605 horses from 48 breeds, has identified more than 32.8 million variant sites and revealed marked differences among breeds in predicted genetic load, defined as the number of potentially deleterious variants and their homozygous occurrences. These metrics show detectable correlations with effective population size and inbreeding history, offering a new quantitative framework for analysing inter‐breed differences in genetic diversity and health risk from the perspective of mutational burden and demographic history (Durward‐Akhurst et al. 2024). Importantly, genetic load should be interpreted together with demographic context, because closed breeding, small effective population size and recent bottlenecks can increase homozygosity of deleterious alleles even when overall breed identity is well maintained. Such information can support evidence‐based mating design, carrier screening and conservation planning by helping breeders reduce harmful allele accumulation while retaining valuable breed‐specific diversity.

### 4.3 Maternal, Paternal and Autosomal Signatures of Diversity

Domestication and subsequent breeding have produced a strikingly asymmetric pattern of genetic diversity in maternal versus paternal lineages. Mitochondrial DNA studies indicate that early domestic horses widely incorporated wild maternal lineages, generating diverse haplogroups and deeply branching maternal phylogenies (Lippold et al. 2011). By contrast, the Y chromosome of modern domestic horses is highly homogenized, reflecting strong control over male reproduction and the historical impact of a limited number of key founding stallions (Wallner et al. 2013, 2017). After establishing a dense map of polymorphic loci on the male‐specific region of the Y chromosome (MSY), researchers reconstructed MSY genealogies and found that the vast majority of modern stallions share very closely related MSY haplotypes, a pattern consistent with intensive use of a few elite stallions and strong directional male‐line breeding over the past two centuries (Wallner et al. 2013, 2017). Large‐scale Y chromosome analyses have more recently confirmed the signal of a dominant paternal lineage with origins in eastern Eurasia and its major role in global dissemination (Radovic et al. 2024). Together, the broad introduction of maternal diversity and the strong constraint on paternal diversity form a defining feature of the present‐day genetic structure of modern domestic horses (Lippold et al. 2011; Radovic et al. 2024; Wallner et al. 2013, 2017).

On autosomes, runs of homozygosity (ROH) are widely used as integrated indicators of inbreeding, population bottlenecks and artificial or natural selection, and have become an important tool for reconstructing population history and identifying selection signals in horses. Multi‐breed datasets show substantial variation among breeds in the length and abundance of ROH, as well as the presence of recurrent ROH‐enriched genomic islands containing candidate genes associated with coat colour (*MC1R*, *ASIP*, *STX17*), body size (LCORL/NCAPG, *ZFAT*, *HMGA2*) and performance and metabolism (*PPARGC1A*) (Grilz‐Seger et al. 2019). In small or geographically isolated populations, such as the feral horses of Sable Island, overall ROH burden is markedly elevated, indicating reduced effective population size and increased inbreeding risk (Colpitts et al. 2022; Grilz‐Seger et al. 2019). Systematic comparisons among six domestic breeds further show that long ROH segments are more informative about recent inbreeding, whereas short ROH segments primarily reflect historical bottlenecks. In practice, these two classes of ROH can thus be used as genetic scales for distinguishing demographic processes operating over different temporal windows (Szmatoła et al. 2022).

### 4.4 Ancient Genomes and the Current Diversity Landscape

Time‐stratified ancient genomes provide direct evidence for how genetic diversity has evolved over long timescales. Analyses of ancient and modern horse genomes spanning roughly five millennia show that the large‐scale lineage replacement occurring after the Bronze Age swept across much of Eurasia, driving modern domestic horses towards broad ancestry sharing across large geographic regions and leaving strong selection signals in pathways related to management regimes and rideability (Fages et al. 2019).

Seen in this light, the present pattern of shared DOM2‐related ancestry across many domestic horses, combined with pronounced regional breed specificity in modern domestic horses, matches well with the domestication and dispersal history reconstructed from ancient genomes. It reflects the combined outcome of multiple lineage replacement events and intense selection under modern breeding. Against this background, genetic diversity conservation and the sustainable use of local breeds must simultaneously account for the robustness of the global ancestral framework and the vulnerability of small local populations. It also provides the population‐genetic foundation for the following sections on key economic traits and health resilience.

## 5 Genetic Dissection of Key Economic Traits in Horses

With high‐quality reference genomes and population datasets now available, equine genomics has increasingly turned to the genetic basis of economically important traits. Recent studies on athletic performance, coat colour, body size, environmental adaptation and inherited diseases/myopathies have linked representative association signals to specific genes and pathways and, in some cases, to experimentally supported mechanisms. As a whole, this body of work illustrates how molecular markers are beginning to inform routine breeding and health management, and how genomic data can be connected more directly to productivity, welfare and on‐farm decision‐making.

### 5.1 Athletic Performance

Racing results, gait and jumping ability are complex performance traits shaped by polygenic effects as well as training and management practices (Hill et al. 2010, 2019). At the level of metabolic pathways, genes involved in mitochondrial respiration and substrate switching (e.g., *COX4I2*, *PDK4*) are significantly associated with endurance versus speed phenotypes. Analyses of muscle biopsies before and after training further show measurable transcriptional responses of these genes in aerobic metabolism and carbohydrate–lipid metabolic programmes (Hill et al. 2010).

With respect to skeletal muscle development and energy mobilization, *MSTN* allelic combinations are strongly associated with preferred racing distance and sprint capacity in Thoroughbreds. Related markers are now widely used for ‘speed gene’ genotyping and training stratification in multiple racing populations, making *MSTN* one of the earliest molecular tools to be implemented in athletic performance profiling (Binns et al. 2010; Hill et al. 2019).

For gait, a key nonsense mutation in *DMRT3* (p.Ser301Ter, commonly referred to as *DMRT3*:Ser301STOP) is very strongly associated with lateral gaits such as pace and ambling, and has been repeatedly validated across multiple gaited breeds. Its frequency distribution and historical diffusion trajectory in different global lineages have been mapped, showing long‐term accumulation and artificial selection for this mutation in gaited lines (Andersson et al. 2012; Kristjansson et al. 2014; Promerová et al. 2014). In China, Chakouyi (CKY) horses, commonly known in Chinese as zouma, are famous for their stable lateral gait, smooth ride and strong environmental adaptability in cold–arid highland regions. Resequencing and selection scans indicate that this population is genetically closer to Kazakh and Mongolian horses, and they pinpoint the *DMRT3* causal mutation g.22999655C>A in the ECA23:22.39–22.41 Mb region. This allele is at high frequency in CKY and matches well with its characteristic gait phenotype (Li et al. 2022; Liu, Fu, et al. 2025).

Genome‐wide association studies for jumping performance indicate that the underlying genetic architecture is not dominated by a single major locus, but instead arises from the combined effects of multiple pathways. Candidate genes include *PAPSS2*, *MYL2*, *TRHR*, *GABPA*, *NRAP* and *TBX4*, which participate in skeletal development, muscle strength regulation and neuroendocrine pathways, together supporting an integrated regulatory framework spanning the bone, muscle and nervous systems (Brard and Ricard 2015; Schröder et al. 2012). In addition, several metabolism‐ and muscle‐related genes (e.g., *CKM*, *COX4I2*) have been associated with racing performance metrics, suggesting tight coupling between metabolic efficiency, muscular power and neural control. This provides a molecular basis for future optimization of performance via combined selection and precision training (Gu et al. 2010; Hill et al. 2019).

Consistent with this integrated bone–muscle–neural framework, the latest time‐series analyses of ancient and modern genomes have detected strong selection at the *GSDMC* locus associated with trunk configuration, spinal morphology and motor coordination. These findings imply that the genetic basis of spinal stability and load‐bearing capacity is also an important component in the emergence of highly rideable horses (Liu, Jia, et al. 2025).

### 5.2 Coat Colour

Equine coat colours can broadly be grouped into three categories: base colours, dilution colours and white spotting/depigmentation. Biologically, they reflect the development and migration of melanocytes, and the synthesis, transport and spatial distribution of eumelanin and pheomelanin (Brooks and Bellone 2013). Base colours are primarily determined by the antagonistic MC1R–ASIP axis: functional *MC1R* alleles favour eumelanin synthesis, whereas *ASIP* antagonizes *MC1R* signalling and shifts pigmentation towards pheomelanin. Haplotypes at these two loci jointly produce the main base coat phenotypes of black, chestnut and bay. This classical two‐locus model has been validated in multiple breeds and supports a stable genotype–phenotype correspondence framework (Rieder et al. 2001; Sakamoto et al. 2017).

In contrast to the relatively simple architecture of base colours, white spotting and depigmentation are typically highly polygenic. A large number of variants in genes such as *KIT*, *MITF*, *PAX3*, *HPS5*, *EDNRB* and *RFWD3* can cause varying degrees of white spotting or depigmentation, with effect sizes ranging from localized facial blazes and sock markings to nearly all‐white individuals. Some alleles are also associated with health risks such as deafness and congenital stationary night blindness (McFadden et al. 2024). In practice, visual assessment of coat colour can be confounded by sun bleaching, age‐related greying, epistasis and allelic combinations. Integrating standardized phenotypic records with molecular genotyping in registration and breeding programmes can therefore improve the consistency and traceability of coat colour classification (McFadden et al. 2024).

Dilution colours (e.g., cream, pearl, champagne) usually reflect reduced synthesis or transport of melanin. Variants in *SLC45A2* are strongly associated with cream and pearl phenotypes and represent major determinants of dilution (Locke et al. 2001). A missense mutation in *SLC36A1* underlies champagne‐like generalized dilution, while *PMEL* (silver) variants primarily affect eumelanin‐based pigmentation and give rise to characteristic silver coats (Brunberg et al. 2006; Cook et al. 2008; Holl et al. 2019). These loci collectively illustrate how, even on the same base‐colour background, modifications to melanin synthesis, loading, or deposition in melanosomes can generate fine‐grained dilution gradients and regional pigmentation patterns.

White spotting and depigmentation can be layered onto any base colour and are often closely related to abnormalities in melanocyte migration, proliferation and survival during embryogenesis. Multiple polymorphisms in *KIT* determine the extent and pattern of spotting and provide a major molecular basis for the spectrum from pinto to near‐white phenotypes (Haase et al. 2007). Pathogenic variants in *MITF* and *PAX3* can produce the characteristic splashed white pattern, whereas deleterious *EDNRB* alleles cause overo lethal white syndrome (OLWS), marked by severe enteric neural crest defects (Hauswirth et al. 2012; McFadden et al. 2023; Metallinos et al. 1998; Santschi et al. 1998). In addition, the grey phenotype is caused by a tandem duplication near STX17 that leads to progressive depigmentation and is associated with a markedly increased risk of melanoma (Pielberg et al. 2008). The dun primitive colour pattern, by contrast, is driven by TBX3‐mediated spatial regulation of pigment distribution across the trunk and limbs, producing the classic dorsal stripe and shoulder barring (Imsland et al. 2016).

Overall, equine coat colour represents not only a set of classical Mendelian traits but also a visually accessible readout of neural crest development, melanocyte biology and interactions within the skin–hair follicle microenvironment, with important implications for breed identification, aesthetic selection and management of genetic disease risk.

### 5.3 Body Size

Body size, especially height at the withers, has high heritability in horses and follows an architecture in which a few major loci act on top of a broad polygenic background (Makvandi‐Nejad et al. 2012; Signer‐Hasler et al. 2012). Large‐sample GWAS have repeatedly identified loci near LCORL/NCAPG, *HMGA2*, *ZFAT* and *LASP1* as significant height‐associated signals across breeds, with a small number of major‐effect loci explaining a substantial proportion of height variation (Makvandi‐Nejad et al. 2012; Signer‐Hasler et al. 2012; Tetens et al. 2013). Among these, a nonsynonymous variant in *HMGA2* shows a clear dosage effect on stature in Shetland ponies and has strong functional support, representing a landmark case in anchoring quantitative height variation to specific molecular mechanisms (Frischknecht et al. 2015).

In Chinese horse populations, a key mutation in an enhancer region on chromosome 8 upregulates *TBX3* expression, promotes distal limb bone growth, accelerates height differentiation and contributes to the formation of graded body‐size variation among breeds (Liu et al. 2022). At the level of regional genomic structure, structural variants in the LCORL/NCAPG neighbourhood have been repeatedly associated with long‐bone growth signals in independent studies, underscoring the central regulatory role of this region in skeletal elongation (Staiger et al. 2016). Considered jointly, population association and functional evidence support a model in which a limited set of major loci, chromatin structural changes and networks of growth factors and transcription factors jointly shape a continuous gradient of body size in horses (Liu et al. 2022; Makvandi‐Nejad et al. 2012; Staiger et al. 2016).

Small stature (ponies) has distinct value in the equine industry and in companion and tourism contexts, but its phenotypic origin is heterogeneous. It can reflect continuous quantitative reduction in height within the normal variation range, or it may result from discrete congenital defects in cartilage and skeletal development pathways (Andrade, Basso, Castiglioni, et al. 2020; Metzger et al. 2017). Clarifying the genetic boundaries between these mechanisms is important both for leveraging small size in breed innovation and for identifying and avoiding recessive pathogenic alleles that pose welfare concerns and breeding risks (Leegwater et al. 2016; Rafati et al. 2016).

To further dissect the genetic basis of pony/dwarf phenotypes, research has identified a number of clearly pathogenic loci that point directly to congenital disturbances in cartilage development and limb bone growth, beyond the height‐associated loci *HMGA2*, LCORL/NCAPG and *TBX3*. For example, multiple deleterious *ACAN* (aggrecan) alleles (D1–D4) have been identified in miniature horses, Shetland ponies and related breeds (Eberth et al. 2018). Individuals homozygous for D4 exhibit classic dwarfism with disproportionate heads and limbs and tarsal/carpal deformities, indicating that *ACAN* multi‐allelism can converge on similar short‐limbed, dwarfed phenotypes via distinct molecular routes (Andrade, Basso, Castiglioni, et al. 2020; Andrade, Basso, Magro, et al. 2020; Eberth et al. 2018).

A splice‐site mutation in *B4GALT7* is responsible for a dwarfism with joint laxity syndrome in Friesians, highlighting the critical role of glycosaminoglycan synthesis and extracellular matrix homeostasis in the columnar organization of growth plate cartilage and the mechanical properties of ligaments. Case–control analyses and functional assays consistently support its pathogenicity (Leegwater et al. 2016). In Shetland ponies, skeletal atavism (SA) is closely linked to large deletions of *SHOX* in the pseudoautosomal region of the sex chromosomes. Clinically, SA is characterized by elongation of the ulna and fibula, incomplete fusion of the radius/ulna and tibia/fibula, and abnormal carpal/tarsal angles. The deletion of the biale SHOX/PAR region (Del‐1/Del‐1, Del‐2/Del‐2, or Del‐1/Del‐2) is a diseased genotype of Shetland pony skeletal atavism, indicating that distal limb skeletal development is highly sensitive to *SHOX* dosage and the integrity of its regulated region (Rafati et al. 2016).

Overall, polygenic quantitative variation in height defines a continuous body‐size gradient from large to small horses, whereas monogenic defects in cartilage development and skeletal morphology set discrete pathological thresholds and breed‐specific risks. Together, these two dimensions refine the genetic map of equine body size and provide actionable molecular targets for carrier testing and mating‐risk management in pony breeds (Andrade, Basso, Castiglioni, et al. 2020; Andrade, Basso, Magro, et al. 2020; Leegwater et al. 2016; Metzger et al. 2017; Rafati et al. 2016).

### 5.4 Environmental Adaptation

Environmental adaptability involves coordinated regulation of thermoregulation, coat shedding and renewal, lipid metabolism, oxygen transport and immune responses (Hoekstra 2006; Orlando 2020). In high‐altitude and cold–arid steppe environments, hypoxia and low temperature make oxygen transport and energy metabolism central adaptive axes. In highland horse populations, oxygen‐sensing pathways centred on *EPAS1* (*HIF‐2α*), together with networks regulating oxygen transport and erythropoiesis, have repeatedly been identified as key candidates. Studies of Tibetan horses show that specific missense mutations in *EPAS1* enhance protein stability and increase its dimerization with *ARNT* (*HIF1B*), thereby amplifying hypoxia signalling outputs under low‐oxygen conditions and establishing a molecular–phenotypic evidence chain that links *EPAS1* variation to blood oxygen transport, erythropoietic regulation and ultimately high‐altitude tolerance (Yue et al. 2019).

From a cross‐species perspective, HIF‐2 (*EPAS1*) is a central regulator of erythropoietin (EPO) and erythropoiesis and has been shown in mouse models to play critical roles in haematopoiesis and cardiopulmonary function, providing strong biological support for the EPAS1‐based adaptation observed in highland horse breeds (Rankin et al. 2007; Scortegagna et al. 2003). As a local breed distributed on the Qinghai–Tibet Plateau and in the Qilian Mountains, CKY horses also display partial overlap with Tibetan horses in their genetic basis of high‐altitude adaptation. In addition to the gait‐related region, *EPAS1* signals associated with hypoxia tolerance are evident in the CKY genome, and CAT has been highlighted as a potential component of oxidative stress and stress‐resistance pathways. Combined with low inbreeding coefficients and relatively high nucleotide diversity, these findings collectively support a long‐term co‐shaping process in which advantages in gait and altitude adaptation have evolved in parallel (Li et al. 2022; Liu, Fu, et al. 2025).

Across broader steppe and cold–arid environments, differentiation in energy metabolism and immune pathways further shapes the ecological adaptation of local breeds. Reference assemblies and population analyses of Mongolian horses and Przewalski's horses have revealed pronounced differentiation in pathways related to energy metabolism and immunity, providing mechanistic support for the endurance and robustness of Mongolian horses (Huang et al. 2014). These findings are consistent with the selection signals at *EPAS1* and antioxidant defence pathways observed in high‐altitude and cold–arid breeds and together outline an adaptive module that underpins performance in hypoxic, cold–arid and high‐workload environments.

In contrast, local breeds in hot–humid regions are characterized more by adaptation along the heat stress–immune defence axis. In Jinjiang horses, for example, genome‐wide variation analyses have detected copy number variants and selection signatures near genes such as *HSPA1A*, *NFKBIA* and *SOCS4*, suggesting that networks involved in cellular protection and inflammatory regulation have been remodelled to cope with heat stress and pathogen pressure in hot–humid climates (Wang et al. 2022).

As discussed above, ancient genomic time series add a deep‐time perspective on how adaptive landscapes have been reshaped. Analyses of ancient and modern genomes spanning roughly five millennia indicate that, after the Bronze Age, signalling and skeletal–muscular pathways related to management practices and rideability experienced sustained intensification of selection, emphasizing the central role of human activities in shaping the adaptive landscape of domestic horses (Fages et al. 2019).

Considered alongside modern breed genomic data, ancient and contemporary evidence converge on a two‐layered view. On the one hand, environmental gradients (high altitude, cold–arid, hot–humid) drive regional adaptation primarily through modules involving *EPAS1*, energy metabolism and immunity. On the other hand, long‐term human selection for riding performance and working capacity is superimposed on these environmental pressures, jointly determining the adaptive genetic architecture of modern domestic horses (Fages et al. 2019; Huang et al. 2014).

### 5.5 Genetic Diseases and Myopathies

Beyond performance‐related traits such as athletic ability, coat colour, body size and environmental adaptation, health and lifespan have a fundamental impact on the usability of horses. Many monogenic diseases not only directly impair performance and welfare but also influence breeding strategies and population structure through the removal or restricted use of affected and carrier animals (Valberg et al. 2023). Among these conditions, myopathies and connective tissue disorders have attracted particular attention. Their molecular bases are now relatively well characterized and are increasingly integrated into management and breeding strategies (McCue, Valberg, Lucio, and Mickelson 2008; Mccue, Valberg, Miller, et al. 2008; Tryon et al. 2007).

Polysaccharide storage myopathy type 1 (*PSSM1*) is one of the best‐studied genetic myopathies. It is caused by a functional variant in *GYS1* that leads to abnormal glycogen accumulation in skeletal muscle and presents clinically with post‐exercise myalgia, stiffness, fasciculations and, in severe cases, rhabdomyolysis (McCue, Valberg, Lucio, and Mickelson 2008; Mccue, Valberg, Miller, et al. 2008). The causative mutation has a particularly high prevalence in Quarter Horses and related draught breeds, and its pathogenicity and population burden have been repeatedly confirmed by molecular diagnostics, pedigree analyses and population surveys (Mccue, Valberg, Miller, et al. 2008; Valberg et al. 2023). Systematic reviews show that *PSSM1* prevalence and phenotypic severity vary among breeds and that there are significant interactions among genotype, dietary composition and training intensity. High‐starch/high‐sugar diets and irregular exercise regimes exacerbate disease risk, whereas limiting non‐structural carbohydrates, increasing dietary fat and implementing moderate, regular exercise can mitigate clinical signs (Valberg et al. 2023).

Hyperkalemic periodic paralysis (HYPP) is tightly associated with a point mutation in the skeletal muscle sodium channel gene *SCN4A*. Episodes are characterized by fasciculations, muscle weakness and even flaccid paralysis, and are often triggered or exacerbated by electrolyte disturbances, especially elevated serum potassium (Rudolph et al. 1992). The mutation exhibits incomplete dominant inheritance, and heterozygous carriers can show a spectrum of clinical signs. Early founder effects and pedigree‐based selection in some performance lines are believed to have facilitated the spread of the HYPP allele (Rudolph et al. 1992; Valberg et al. 2023). Hereditary equine regional dermal asthenia (HERDA), by contrast, is caused by a missense mutation in *PPIB* that impairs collagen maturation. Clinically, affected horses present with excessively loose, fragile skin over the back and withers and poorly healing wounds. The disease is inherited recessively, and matings between carriers are the primary source of affected foals (Tryon et al. 2007).

Another myopathy reported in Quarter Horse foals—Glycogen Branching Enzyme Deficiency (GBED)—is a lethal glycogen storage disease (Wagner et al. 2006). Affected foals may present with stillbirth, neonatal weakness, seizures, or respiratory or cardiac failure, and often succumb early in life (Valberg et al. 2001). Studies have demonstrated that GBED is caused by a nonsense mutation (c.102C>A; p.Y34*) in the *GBE1* gene; this mutation occurs at a certain carrier frequency within Quarter Horse and Paint Horse populations (Ward et al. 2003). Consequently, GBED constitutes a significant autosomal recessive lethal genetic disease among Quarter Horse‐related breeds; therefore, genotyping individual horses to avoid breeding between carriers is a critical measure for mitigating the risk of this disease.

For the three diseases mentioned above, robust molecular tests have been developed, and carrier screening combined with mating restrictions has proven effective in reducing the incidence of new cases. These conditions provide clear examples of how molecular diagnostics can be embedded directly into breeding decisions in the equine industry (Valberg et al. 2023).

In addition, MYH1‐associated immune‐mediated myositis/myopathy is closely linked to specific alleles and is characterized by acute muscle atrophy, markedly elevated muscle enzymes (e.g., CK), and recurrent episodes of weakness and exercise intolerance (Finno et al. 2018). Case–control studies and clinical follow‐up indicate that this condition is best understood within a framework that combines inherited genetic susceptibility with environmental triggers such as vaccination, infection or stress, making it a typical example of a gene–environment interaction myopathy (Finno et al. 2018; Gianino et al. 2019). Management and prevention of such diseases rely on both genetic testing to identify high‐risk genotypes and adjustments to immunization schedules, training and husbandry practices to reduce triggering events (Finno et al. 2018; Valberg et al. 2023).

Overall, genetic studies of PSSM1, HYPP, HERDA and MYH1‐associated myopathies now provide a coherent chain of evidence from causal variants to population burden. Embedding these markers into routine breeding and health management—for example, via stallion screening, controlled mating and tailored feeding and training programmes—offers a practical route to reduce genetic disease while maintaining or improving performance and welfare (Finno et al. 2018; Gianino et al. 2019; Valberg et al. 2023). For ease of overview and cross‐trait comparison, Table 2 summarizes representative candidate genes, major pathways and applied implications for the five categories of key economic traits discussed above.

**TABLE 2:** Candidate genes and pathways underlying major economic traits in horses.

Traits category	Representative traits	Key genes/loci	Main pathways/mechanisms	Breeding/application	Key references
Athletic performance	Speed, stamina, gait, jumping	*MSTN*, *DMRT3*, *COX4I2*, *PDK4*	Muscle development; energy metabolism; gait control	Genotyping for speed/gait typing and stallion selection	Hill et al. (2019); Liu, Fu, et al. (2025); Wang et al. (2024)
Coat colour	Base colours, dilution, white/grey, dun	*MC1R*, *ASIP*, *KIT*, *STX17*, *PMEL*, *MITF*	Melanocyte biology; eumelanin/pheomelanin balance; melanosome transport	Colour registration and targeted colour breeding; screening risk alleles (e.g., OLWS, grey)	Holl et al. (2019); McFadden et al. (2023, 2024)
Body size & skeletal development	Height gradient; large–small size continuum; pony/dwarf phenotypes	LCORL/NCAPG, *HMGA2*, *TBX3*, *ACAN*, *SHOX*	Growth‐factor and TF networks; enhancers/chromatin; cartilage ECM; limb development	Separate normal height variation from skeletal defects; carrier testing and mating design	Andrade, Basso, Castiglioni, et al. (2020); Andrade, Basso, Magro, et al. (2020); Liu et al. (2022)
Environmental adaptation	High‐altitude/cold–dry; hot–humid	*EPAS1*, *CAT*, *PPARGC1A*, *HSPA1A*, *NFKBIA*	HIF–EPO axis; cardio‐pulmonary and metabolic remodelling; stress and immune responses	Markers for regional breeding, conservation and climate‐resilient deployment	Li et al. (2022); Liu et al. (2019); Wang et al. (2022)
Genetic diseases & myopathies	PSSM1, HYPP, HERDA, immune‐mediated myositis	*GYS1*, *SCN4A*, *PPIB*, *MYH1*	Glycogen storage; Na^+^ channels; collagen/ECM; immune‐mediated myositis	Integrate genetic tests into breeding and health plans; avoid high‐risk matings	Finno et al. (2018); Gianino et al. (2019); Valberg et al. (2023)

## 6 Conclusions and Perspectives

By integrating ancient DNA with modern population genomic data, the genetic landscape of contemporary domestic horses can be viewed along two tightly coupled axes. Deep‐time lineage divergence, a Bronze Age wave of unified replacement and strong artificial selection under modern breeding regimes have together shaped a largely homogenised pan‐Eurasian ancestry backbone. At the same time, mitochondrial DNA, Y‐chromosomal variation and autosomal runs of homozygosity reveal pronounced contrasts between diverse maternal and highly constrained paternal lineages, as well as inter‐breed differences arising from inbreeding, bottlenecks and selection intensity. These features provide the key population‐genetic backdrop for interpreting breed formation and evaluating health risks.

On this basis, genetic improvement in horses can be organised along a pathway that links genomic resources, population structure, functional interpretation and practical application. High‐quality reference genomes and dense population datasets make it possible to capture complex variation by jointly considering SNPs, structural variants, runs of homozygosity, mtDNA and MSY when reconstructing population histories and mutational burdens. Around key economic traits, GWAS, selection scans and functional assays can be combined to build evidence chains from variants through pathways to phenotypes, and molecular markers emerging from these studies can be incorporated into breeding and health‐management pipelines, for example in stallion selection, mating schemes and inbreeding monitoring.

Future progress is likely to come from several directions rather than a single technical breakthrough. Extending telomere‐to‐telomere assemblies and pangenome resources to locally adapted and endangered breeds, and treating structural‐variant detection as a routine rather than specialised option, should help capture the full spectrum of equine genomic diversity. At the same time, closer integration between population‐level association studies and multi‐omics or model‐based functional experiments will narrow the gap between locus discovery and mechanistic insight. A third, more applied strand concerns the economics and logistics of using runs of homozygosity, parental haplotypes and key trait markers in germplasm evaluation and health surveillance in real breeding programmes. In combination, these lines of work can strengthen the links between resource conservation, health management and molecular breeding, and may also offer a useful template for comparative genomics and adaptive evolution research in other domestic animals and their wild relatives.

## Funding

This work was supported by grants from ‘Xingdian Talent’ Industry Innovation Talent Program in Yunnan Province (XDYC‐CYCX‐2022‐0029).

## Ethics Statement

The authors have nothing to report.

## Consent

The authors have nothing to report.

## Conflicts of Interest

The authors declare no conflicts of interest.

## Data Availability

The authors have nothing to report.
